# No evidence for multiple loci affecting rheumatoid arthritis risk on chromosome 6p21

**DOI:** 10.1186/1753-6561-1-s1-s42

**Published:** 2007-12-18

**Authors:** Richard Sherva, Lingwei Sun, Joanna Biernacka, Rosalind Neuman

**Affiliations:** 1 Department of Psychiatry, Washington University School of Medicine, 660 South Euclid Avenue, St. Louis, Missouri 63104, USA; 2 Institute of Human Genetics, Newcastle University, International Centre for Life, Central Parkway, Newcastle upon Tyne, NE1 3BZ, UK

## Abstract

The influence of certain alleles of the HLA-DRB1 locus on risk for rheumatoid arthritis has been well established through linkage and association studies. In addition, other loci in the HLA region on 6p21 may also affect an individual's risk profile. Here, we used a method to detect excess identity-by-descent sharing between affected sib pairs conditional on the observed genotypes at the hypothesized causal locus to test for the presence of additional arthritis risk loci in the linked region. We used affected sib pairs from two different studies. Because the test depends heavily on specifying accurate allele frequency estimates at the proposed causal locus, we used HLA-DRB1 allele frequency estimates from a large, population-based sample. We also discuss an alternate form of the test in which we could condition on parental genotypes, thereby eliminating the need for actual allele frequencies. The test showed no evidence for the presence of additional arthritis risk loci in the region in the British or North American samples made available for Genetic Analysis Workshop 15. Given the prior knowledge that there likely are arthritis risk loci other than HLA-DRB1 in the region, it appears the tests may have inadequate power to detect the presence of these loci in certain cases.

## Background

There is substantial evidence from linkage and association studies for a locus contributing to rheumatoid arthritis (RA) risk on chromosome 6p21. The HLA-DRB1 locus may be the sole cause of this linkage signal, but given the complexity of the HLA region and autoimmune function, other polymorphisms in the region could also influence RA risk. In addition, previous research suggests the presence of other RA risk loci in the region [1-3]. The analysis presented in this paper uses the method proposed by Sun et al. [4] to test the null hypothesis that HLA-DRB1 genotype is the sole cause of the observed linkage signal.

## Methods

For these analyses we used data from two RA studies, the North American Rheumatoid Arthritis Consortium (NARAC), and The Arthritis and Rheumatism Council's UK National Repository of family material. Details about the designs of these studies are published elsewhere [5-7], but briefly, the NARAC investigators recruited and genotyped families with two or more siblings with RA, and the UK repository pooled a cohort of Caucasian, RA-affected sibling pairs (ASPs).

We initially used Merlin [8] to confirm the RA linkage signal on chromosome 6 with a nonparametric linkage (NPL) statistic [9] in the NARAC and UK data. We also confirmed the association between HLA-DRB1 genotype and RA in the NARAC data set using correlated data corrected logistic regression in SAS PROC GENMOD with HLA-DRB1 modeled as the number of "high-risk" alleles (0, 1, or 2). High-risk alleles were identified based on the work of Newton et al. [10]. This analysis could not be performed in the UK data because all genotyped sibs are affected. An Armitage trend test [11] in SAS Genetics was used to test for association between SNPs in the region and RA. Linkage disequilibrium between the HLA-DRB1 locus and surrounding markers was estimated using MIDAS .

We used the method described by Sun et al. [4] to determine whether the HLA-DRB1 locus is the sole cause of the observed linkage in the region. The test is designed to detect excess identity-by-descent (IBD) sharing between ASPs conditional on the observed genotypes at the hypothesized causal locus. The method is based on the fact that under the null hypothesis that the candidate marker is the sole causal site in the region,

Pr_H0_(I|G_C_, ASP) = Pr(I|G_C_),

where Pr_H0 _is the probability that the hypothesized causal marker is the sole cause of the linkage signal, I is the IBD sharing for a sib pair at the candidate locus, and G_C _indicates the sibs' genotype configuration at the locus. Sun et al. use the distribution of IBD sharing between affected sibs given the sibs' genotypes at the candidate locus, G_C_, (which depends on allele frequencies at the candidate locus) to obtain the null conditional mean sharing, μ_G_, which is equal to E_H0_[S], and variance, σ_G_^2^, which is equal to Var_H0_[S] for some IBD sharing statistic S. A variation of the usual NPL score statistic or the linear or exponential likelihood of Kong and Cox [9] based on the standardized family score statistics Z = {S - μ_G_}/σ_G _is then used to assess evidence against H_0_. We used population allele frequencies from published results by Klitz et al. [12] from a study of 1000 randomly selected North American Caucasian donors. When multiple ASP were present in a family, one was randomly chosen from each family in the NARAC and UK data set for this analysis, and pairs with informative genotypes (at least one shared DRB1 allele) were given weights based on σ_G_. To obtain the actual sharing between sibs, we used Merlin [8] to calculate IBD probabilities at the DRB1 locus based on DRB1 genotype as well as the genotypes at flanking markers. To compute S, we summed the products of the probabilities and the number of alleles shared IBD.

We also considered a modified version of the method of Sun et al. proposed by Biernacka et al. [13]. In this modified method, conditioning on the parental genotypes avoids the problem having to specify allele frequencies in the analysis. In the modified method, G_C _in Eq. (1) is replaced by the parental and ASP genotypes, {G_P_, G_C_}, and μ_G _and σ_G _are based on the IBD distribution given the ASP and parental candidate SNP genotypes.

## Results

Using both the NARAC and UK data, we confirmed the evidence for linkage to RA on chromosome 6. The peak LOD score in NARAC families was at marker D6S1629 (47.7 cM, LOD = 14.36, *p *= 4.28 × 10^-16^), and the peak score in the UK families was at marker D6S276 (44.4 cM, LOD = 4.28, *p *= 9.01 × 10^-6^). We also confirmed the association of HLA-DRB1 alleles with RA in the NARAC data set. In NARAC families, the odds ratio for each additional risk allele was 18.2, (*p *< 0.0001). One SNP, rs910516, is located approximately 5 Mb from D6S1629 and suggests association with RA in the NARAC data (*p *= 0.01). There is significant LD between D6S1629 and HLA-DRB1 alleles; only one high-risk allele (1402), however, is in significant LD with an allele (3) of D6S1629 (D' = 0.64).

The Sun et al. test in the HLA-DRB1 region, computed in 452 Caucasian ASPs from the NARAC data failed to reject the null (T = -0.2, *p *= 0.58), suggesting that the HLA-DRB1 locus may be the sole contributor to the linkage signal. We got a similar result in the 309 ASP from the UK data (T = -0.6, *p *= 0.73). Table 1 summarizes the results from the analyses in both data sets.

**Table 1:** Results from linkage and association tests between DRB1 and RA, and for oversharing on 6p21

	Sample	
	
	NARAC (452 ASP)	UK (309 ASP)
LOD score at DRB1 locus (*p*-value)	14.36 (*p *= 4.28 × 10^-16^)	4.28 (*p *= 9.01 × 10^-6^)
RA odds ratio for each DRB1 high-risk allele (*p*-value)	18.2 (*p *< 0.0001)	N/A^a^
Sun method T statistic (*p*-value)	T = -0.2 (*p *= 1)	T = -0.6 (*p *= 1)

^a^Not applicable because all genotyped individuals in the UK sample are affected.

## Discussion

The results of our analyses provided no evidence for the presence of genetic sharing in excess of what could be explained by the DRB1 locus, making it unclear whether DRB1 is the sole RA causal locus in the HLA region on chromosome 6. The analyses may have had insufficient power to detect the effects of additional loci near the DRB1 locus. First, the samples used in these analyses are not particularly large. More importantly, the method of Sun et al. is known to have low power for highly informative candidate loci [4]. The fact that the DRB1 locus is highly polymorphic decreases the power of the Sun et al. test statistic due to the fact that power depends highly on the value of E_A_[S|G] - E_H0_[S|G], where E is the conditional expected value under the true genetic model, S is the sharing statistic, and G is the siblings' genotype configuration at the hypothesized causal locus. When G provides close to complete information on S, as is more likely with a highly polymorphic marker, power to detect sharing in excess of that due to the hypothesized causal locus is low. In the simulated Genetic Analysis Workshop 15 data, Biernacka et al. found that their modified version of the Sun method had little power to detect effects of additional loci after accounting for the effect of DRB1 [13]. For a detailed discussion of factors that affect the power of the Sun method, see [4].

We did a simple calculation to determine the effect size that would have allowed us to reject H_0 _in the NARAC data. Using the actual null conditional variances of the observed allele sharing in the sibs, we would need an average difference between the actual and null conditional sharing of 0.04 across the 452 ASP to reject H_0 _at *p *= 0.05. In the NARAC families, only 178 out of 452 selected ASP provide evidence against H_0_. The remaining pairs show less sharing than expected under the null, and therefore diminished the magnitude of the overall test statistic.

We also considered a modified version of the Sun et al. method proposed by Biernacka et al. [13]. Although too few parental genotypes were available in this data set to make direct comparisons between methods, this approach might be useful in situations in which parents are genotyped and no reliable population allele frequency estimates are available.

It should also be noted that the coding system used for HLA-DRB1 alleles in this data set was not identical to that used in other published literature. Several alleles found in these families were coded differently than in published literature and more refined typing was performed in some families than in others. In these cases, alleles had to be combined into broader categories to make use of the published [12] allele frequencies. This has implications for our results because the accuracy of sharing information is diminished when alleles are combined. Clarification and/or consistency in the genotyping methods and allele coding used would be needed to make full use of the data.

## Conclusion

Given the previous evidence suggesting additional RA risk loci on 6p21, it appears that the Sun et al. test for oversharing may be underpowered to detect additional genetic effects in the region containing the DRB1 locus in these data.

## Competing interests

The author(s) declare that they have no competing interests.
